# Fecal microbiota transplantation—could stool donors’ and receptors’ diet be the key to future success?

**DOI:** 10.3389/fgstr.2023.1270899

**Published:** 2023-11-01

**Authors:** Rita Silva, Liliana Dinis, Arnau Peris, Luís Novais, Conceição Calhau, Diogo Pestana, Cláudia Marques

**Affiliations:** ^1^ Nutrition & Metabolism Department, CINTESIS@RISE, NOVA Medical School, Faculdade de Ciências Médicas (NMS|FCM), Universidade Nova de Lisboa, Lisboa, Portugal; ^2^ YourBiome®, Évora, Portugal; ^3^ CHRC—Comprehensive Health Research Centre, NOVA Medical School, Faculdade de Ciências Médicas (NMS|FCM), Universidade Nova de Lisboa, Lisboa, Portugal; ^4^ LNMD—Laboratory of Neurogastrenterology and Motility, Lisboa, Portugal

**Keywords:** diet, fecal microbiota transplantation, FMT receptors, gut microbiota, stool donors

## Abstract

Fecal microbiota transplantation (FMT) is indicated in many countries for patients with multiple recurrences of *Clostridioides difficile* infection (CDI) for whom appropriate antibiotic treatments have failed. Donor selection is a demanding and rigorous process in view of the implementation of FMT programs worldwide. One of the most noteworthy factors that has been shown to affect FMT outcomes is the microbial diversity of the stool donor. A detailed assessment of the donor’s microbiota is crucial, as the microbiota is complex, dynamic, and resilient, and a healthy microbiota has several dimensions in addition to the absence of pathogens. Diet is one of the most important factors that modulates the composition and function of the gut microbiome (GM) and has a critical role in orchestrating the host–microbiota crosstalk throughout life. The diversity of the human GM seems to be related to variations in dietary patterns. Currently, the dietary patterns of stool donors and receptors are not taken into consideration in any way for FMT. In this study, we reflect on the importance of including this type of assessment in the stool donor screening process and knowing the impact of diet on the GM, as well as the importance of monitoring receptors’ diet to ensure the engraftment of the transplanted microbiota.

## Introduction

The gut microbiome encodes over 3 million genes, whereas the human genome consists of approximately 23,000 genes (1). Therefore, the metabolic capacity of the gut microbiome greatly exceeds the metabolic capacity of human cells (2). The gut microbiota (GM) has a crucial role in the maintenance of health, with protective, structural, and metabolic functions (3). An imbalance in its composition and function (dysbiosis) has been associated with many disorders (4), including *Clostridioides difficile* infections (CDIs).

Fecal microbiota transplantation (FMT) was first described in the fourth century by the traditional Chinese medicine doctor Ge Hong (5), but it was only in 1983 that Schwan et al. published the first report of a successful treatment with FMT for CDI, through retention enema (6). Currently, FMT is an established treatment for recurrent CDI (7, 8), but it also seems promising as a therapy for many other disorders (9).

## Fecal microbiota transplantation

**Figure 1:**
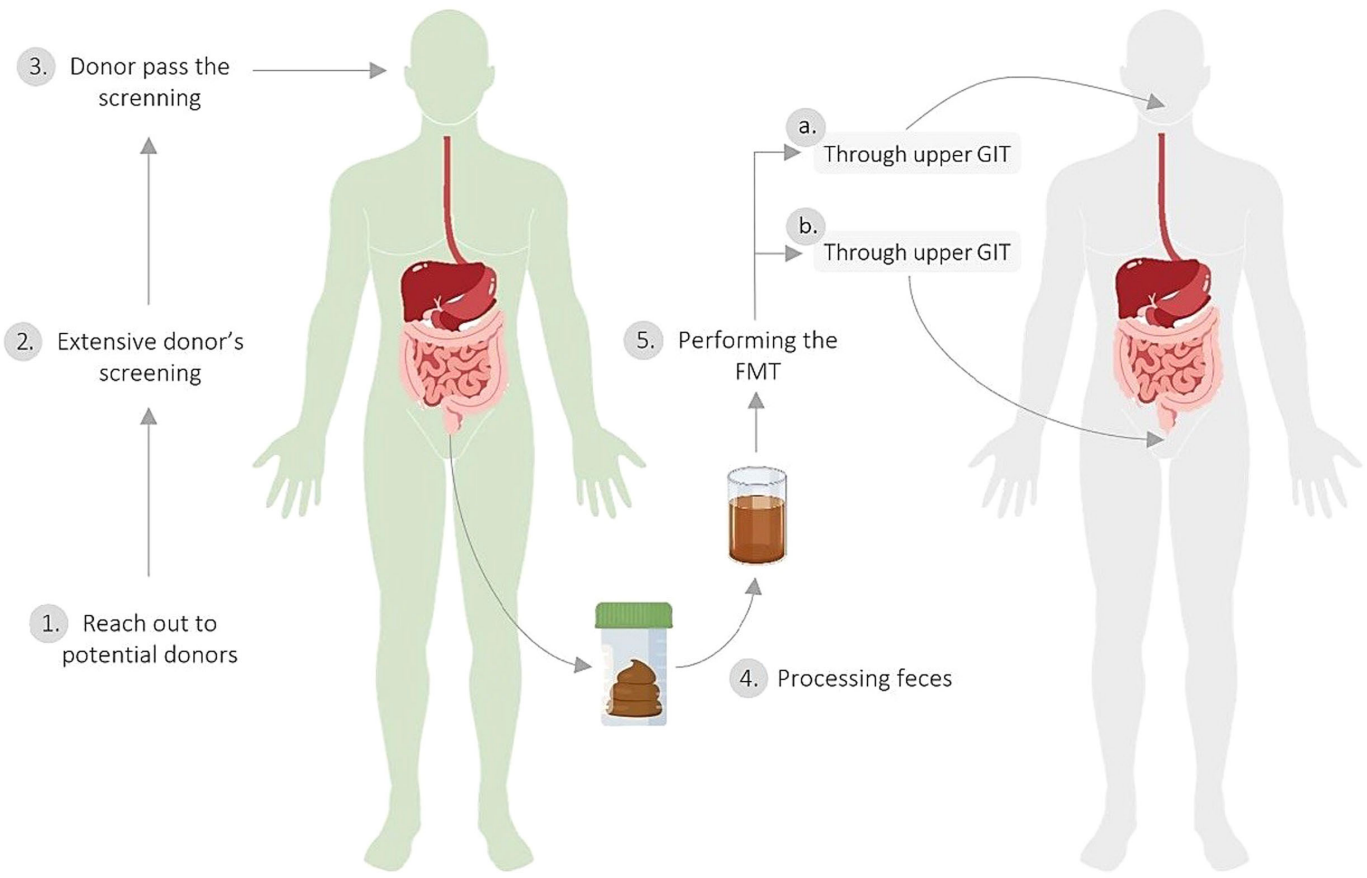
Fecal microbiota transplantation process. 1. Recruitment of potential healthy donors through media and advertising. 2. Extensive donor screening for medical history, infectious diseases, intestinal health, and risk behaviors; blood and stool testing; and screening of stool and stool donors for the presence of SARS-CoV-2 infection. 3. Donor passes the screening. 4. Processing feces through dilution (with 0.9% NaCl) and filtration to obtain microbiota. 5. Performing the FMT **(A)** through the upper GIT, through a duodenal tube or capsules taken orally or **(B)** through the lower GIT, through colonoscopy or an enema. The image is an adaptation of Bou Zerdan M*. et al.* (11), Alabdaljabar et al. (12), and Ooijevaar et al. (9).

FMT is a procedure in which the fecal microbial content from a healthy donor is administered into another patient’s intestinal tract, with the aim of treating a certain disease linked with the alteration of the GM (10). FMT can be performed through the upper gastrointestinal tract (GIT), via a duodenal tube or capsules taken orally, or through the lower GIT, via colonoscopy or an enema (9) (**Figure 1**).

FMT is indicated in many countries (7, 8) for patients with multiple recurrences of CDI for whom appropriate antibiotic treatments have failed (7), and it has cure rates of 80%–90% (13). In addition, it seems promising as a treatment for many other conditions (9). FMT has been studied in inflammatory bowel disease (14), obesity, and metabolic syndrome (15). FMT also seems promising in oncology (16), it might be useful in the prevention and treatment of psychiatric illnesses (17), and has the potential to treat Alzheimer’s and Parkinson’s diseases (18). More recently, it has been proposed as a potential treatment for COVID-19 (19).

### Stool donor screening and stool receptor follow-up

Donor selection is a demanding and rigorous process in view of the implementation of FMT programs worldwide (20). In fact, choosing the right donor could be challenging in clinical practice because of the absence of a clear definition of a healthy GM, and because of the complexity of the host response (such as the immune response) and dietary habits (2).

Potential stool donors should undergo a detailed questionnaire including medical history, infectious diseases, intestinal health, and risk behaviors (21). They should also undergo blood and stool tests to prevent the direct transmission of infectious diseases and avoid transferring an adverse microbiota profile that could possibly increase the risk of the receptor developing other diseases related to an abnormal GM (21, 22).In addition, the US Food and Drug Administration (FDA) has recommended, since March 2020, the screening of stool and stool donors for the presence of SARS-CoV-2 infection (23).

In recent years, the debate about stool donor screening has become deeper. In fact, donors whose stool results in substantially more successful FMT outcomes than the stool of other donors have been described as “super-donors” (24). One of the most noteworthy factors that has been shown to affect FMT outcomes is the microbial diversity of the stool donor (25).

In addition, the stool receptors’ follow-up is focused on the side effects or complications of FMT in the short term (10, 21), and their long-term follow-up includes only the documentation of clinical details and relevant clinical results beyond the first 24 h (21). It does not take into consideration what the receptor should do to keep the transplanted microbiota in balance.

### The importance of the donor’s microbiota

The gut microbiome is complex, dynamic, and resilient, as any biological system (26), and a healthy microbiota has several dimensions in addition to the absence of pathogens. So it is hard to define what a healthy human GM at an exact taxonomic level (27). However, a high level of taxa diversity, a high level of microbial gene richness, and stable microbiome functional cores indicate healthy GM communities (28).

It has been demonstrated that low levels of bacterial gene richness leads to increased adiposity, insulin resistance and dyslipidemia, and a further pronounced inflammatory phenotype (2), indicating the impact of GM on metabolic processes. Besides, an increasing number of diseases are linked with intestinal dysbiosis, such as metabolic, cardiovascular, and neurologic diseases (4), and pathologies such as inflammatory bowel (29) and autoimmune diseases (30). Individuals with these pathologies should not be stool donors, to prevent the transmission of a dysbiotic microbiota that could itself cause the disease in the receptor. Furthermore, donors are excluded based on disease, as we assume that because they have the disease they have an altered GM, but a question remains unanswered: what about those who already have an altered GM and do not have the pathology yet? We propose that the exclusion of these types of donors start to be considered.

### Impact of diet on the gut microbiota

Diet is one of the most important factors that modulates the composition and function of the GM and has a crucial role in orchestrating the host–microbiota crosstalk throughout life (31).

What we eat is a key factor in the composition of the GM, as diet is thought to explain about 20% of microbial structural variations in humans, indicating the ability of dietary approaches to aid in disease management through GM modulation (32). The integration between the GM, food groups, and short-chain fatty acid (SCFA)-producing bacteria is promising in the quest to further upgrade and transform dietary habits (33).

**Figure 2:**
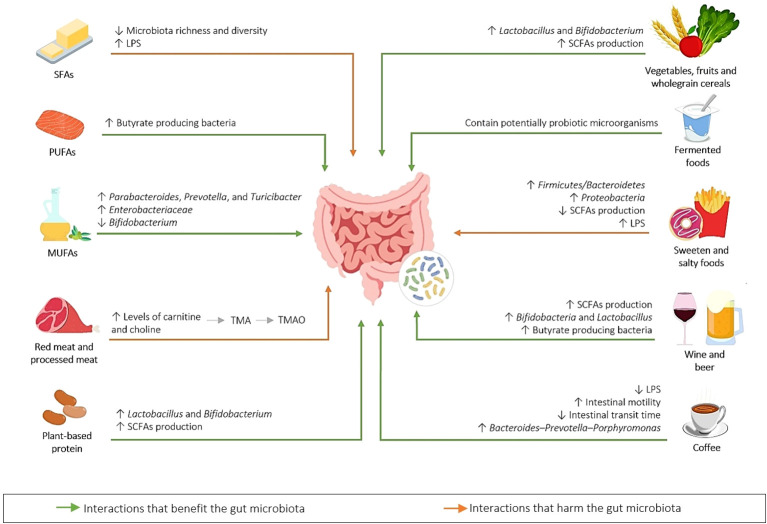
Gut microbiota–diet interactions. Common dietary components are metabolized by the GM to produce metabolites (for example, red and processed meat, containing high levels of carnitine and choline, both of which are precursors that the gut bacteria use to produce trimethylamine (TMA), which is converted by the enzyme flavin-containing monooxygenase 3 (FMO3) into trimethylamine *N*-oxide (TMAO) (34), that has been associated with atherosclerosis). A diet rich in saturated fatty acids (SFAs), sweet and salty foods modify the GM, causing elevated levels of lipopolysaccharides (LPSs) in the circulation, leading to a pro-inflammatory state (metabolic endotoxemia) (35). Some foods have a positive effect on the GM, for example, those that elevate short-chain fatty acid (SCFA) production and the abundance of *Lactobacillus* and *Bifidobacterium* (34), and those that are included in the Mediterranean diet, such as olive oil (35). Fermented foods (36), wine and beer (37), and coffee (38) consumption also have a positive effect on the GM composition. SFA, saturated fatty acid; MUFA, monounsaturated fatty acid; PUFA, polyunsaturated fatty acid; LPS, lipopolysaccharide; SCFA, short-chain fatty acid; TMA, trimethylamine; TMAO, trimethylamine *N*-oxide.

A diverse diet, especially in the number of different types of plant foods eaten, has been linked with greater microbial alpha-diversity, and is thought to enhance the diversity of substrates for the proliferation of numerous taxa (32). The interactions between diet and the GM are described in **Figure 2**.

The diversity of the human GM seems to be related to variations in dietary patterns (39). In fact, several studies have demonstrated the ability of the Mediterranean diet (MD) to modulate the GM and host’s health (40–42). The MD is characterized by a high level of polyphenol-rich product content (extra-virgin olive oil, red wine, vegetables, grains, legumes, whole-grain cereals, and nuts), a positive fatty acid profile [high levels of monounsaturated fatty acids (MUFAs) and polyunsaturated fatty acids (PUFAs), and low levels of saturated fatty acids (SFAs)], and a low intake of processed meat and refined sugars (43). Adherence to the MD was found to be related to increased levels of SCFAs (acetate, propionate, butyrate, and lactate) (3, 42), *Prevotella*, and fiber-degrading *Firmicutes* (44, 45). It is worth mentioning that SCFAs are used as energy sources and participate in numerous metabolic pathways, including gluconeogenesis and lipogenesis, hence contributing to whole-body energy homeostasis (2). The MD has also been linked to improvements in the diversity and richness of the GM (35). Conversely, the Western diet, high in total fat, animal proteins, processed food, refined sugars, and food additives, leads to a dysbiosis in GM composition and is connected with obesity and other metabolic disorders (35).

## Discussion

### Future prospects for dietary screening of stool donors and receptors’ follow-up

Currently, the dietary patterns of stool donors and receptors are not taken into consideration in any guidance for FMT. In addition, no clinical practice recommendations are available to provide receptors or stool donors with dietary advice for FMT (46). Clancy et al. reported that, overall, health professionals and researchers who work with FMT reported that diet was a significant consideration for FMT receptors and donors, and that it would affect the outcomes of the FMT (46). Although they did not usually advise patients to see a dietitian/nutritionist before or after the FMT, and did not feel certain in giving dietary guidance, or that there was enough evidence to provide dietary counsel (46). Owing to the great contribution of diet to the composition and modulation of the GM (43), we consider it crucial to include this step in stool donor screening protocols, in order to guarantee the better quality of the transplanted microbiota and consequent benefits to the host. Moreover, the dietary follow-up of the stool receptors should also be taken into consideration, in order to guarantee the long-term efficiency of the FMT. This assumes a greater importance when FMT is intended to treat metabolic diseases. A study conducted by our group (data not published) showed—through the application of the Mediterranean Diet Adherence Screener (MEDAS) (47), a validated questionnaire to assess adherence to the MD—that only 55.6% (25 out of 45) of the potential stool donors had a level of high adherence (≥ 10) to the MD. These data suggest that the absence of chronic diseases may not be a suitable criterion for donor selection and that diet, as well as other lifestyle factors, should be evaluated to increase FMT efficiency and applicability in health. In addition, recent studies have demonstrated the importance of dietary habits, in particular fiber intake, in optimizing the success of FMT in the treatment of metabolic diseases (48–50). One study used autologous FMT to prolong the beneficial effect of a modified MD on weight regain. The findings of this study provided a provocative perspective where the co-supplementation of low-fermentable fiber may increase the potency of FMT (50). Mocanu et al. also provided a proof of concept for the use of a single-dose oral FMT combined with daily low-fermentable fiber supplementation to improve insulin sensitivity in patients with severe obesity and metabolic syndrome (49). Considering these data, would it not be important to evaluate and, if necessary, modify the dietary habits of the FMT receptor in future protocols?

## Concluding remarks and perspectives

Knowing the impact of diet on the GM, we propose that potential stool donors undergo dietary screening, to increase the probability of a beneficial and functional fecal microbiota being transplanted. We also believe that specific guidelines for stool receptors should be developed, mainly in the treatment of diseases other than CDI.

In addition, besides the inclusion of a dietary screening tool for the stool donor candidates it could be interesting to add nutrition counseling a few months prior to the stool donation in order to improve the quality of the stool donated, if necessary. Nutritionists could also enhance long-term FMT success by giving nutrition counseling services as part of multidisciplinary health care groups (51), as a healthy diet provides the commensal microbes with the substrates necessary for their proliferation and survival (24).

We hope to stimulate future research so that further information about dietary habits of FMT receptors and stool donors can be collected and thus make it possible to better understand the relationship between diet and FMT results. With this information, it would be possible to create a score for stool donors, where diet and other factors that modulate the GM are included. In fact, a validation study of a score of this kind would be interesting to assess whether or not those with the highest scores are, in fact, better donors.

The increased application of FMT in clinical practice will notably have a key impact on public health, as the prevalence of chronic diseases continues to increase. Therefore, the development of FMT protocols that honor this scientific evidence and promote the creation of more detailed screening and follow-up processes are of the utmost importance.

## Data availability statement

The original contributions presented in the study are included in the article/supplementary material. Further inquiries can be directed to the corresponding author.

## Author contributions

RS: Writing – original draft. LD: Writing – review & editing. AP: Writing – review & editing. LN: Writing – review & editing. CC: Writing – review & editing. DP: Writing – review & editing. CM: Writing – review & editing.
